# Editorial: Neurobiological Perspectives in Behavioral Addiction

**DOI:** 10.3389/fpsyt.2019.00003

**Published:** 2019-01-22

**Authors:** Jung-Seok Choi, Daniel Luke King, Young-Chul Jung

**Affiliations:** ^1^Department of Psychiatry, SMG-SNU Boramae Medical Center, Seoul, South Korea; ^2^Department of Psychiatry, Seoul National University College of Medicine, Seoul, South Korea; ^3^School of Psychology, The University of Adelaide, Adelaide, SA, Australia; ^4^Department of Psychiatry, Yonsei University College of Medicine, Seoul, South Korea

**Keywords:** neurobiology, neurocognition, neurophysiology, neuroimaging, behavioral addiction

Some classes of behaviors, including gambling, Internet gaming, and sexual behaviors, may lead to compulsive engagement for a minority of individuals. In extreme cases where individuals may feel unable to control these behaviors without external influence, these behaviors may be considered non-substance or behavioral addictions. Many such behaviors may occur predominantly online, such as gaming, social media, shopping, and pornography, and may be driven by constant accessibility via smartphone and other mobile device technologies. The diagnostic criteria for gambling disorder and Internet gaming disorder (IGD) in the DSM-5 are similar to substance use disorder, referring to symptoms of withdrawal and tolerance, continued use despite negative consequences, and loss of control over the activity. However, some behaviors such as compulsive buying and compulsive sexual behaviors do not have specific diagnostic categories in DSM-5. Many of these behaviors, including emerging online behaviors, will continue to be the subject of discussion among international authorities, such as the World Health Organization (WHO), including calls for more research evidence on behavioral addictions. This Research Topic presents diverse papers on neurobiological evidences of behavioral addictions, encompassing gambling disorder, Internet-based disorders, including Internet gaming disorder and smartphone addiction, and compulsive sexual behaviors.

## Neurobiological Mechanism Underlying Behavioral Addictions

The field of behavioral addictions is continually seeking to identify and understand the important neurobiological mechanisms that drive repetitive, maladaptive behaviors. Animal studies of substance addiction, for example, may help to guide research on the neurobiological mechanisms underlying behavioral addictions. Alcohol withdrawal-anxiety syndrome refers to symptoms that have been shown to depend on molecular and cellular adaptations that lead to persistent, long-term plastic changes in transcription, translation, and synaptic morphology. However, the molecular mechanism underlying the anxiogenic effects of ethanol withdrawal require further study. In the first paper in this Topic, Hou et al. reported that changes in synaptic ultrastructure may be associated with withdrawal anxiety in alcohol dependence.

Impulsivity is considered an important feature associated with the development of addictions. Cho et al. used a rodent version of the gambling task (rGT) to examine how impulsive action and impulsive choice are differentially influenced by difference in age at exposure (i.e., late adolescents/young adults vs. mature adults) to rGT in rats. The results indicated that impulsive action and choice are distinct aspects of impulsivity, which are differentially influenced in rats by the age at the first exposed to gambling task.

One of the major neural networks that play a crucial role in behavioral addiction is the salience network, which mediates the “switching” between neural networks to guide appropriate responses. Alterations in the salience network have been implicated in directing aberrant salience to stimuli associated with addiction, resulting in craving and impaired control over addictive behaviors. Wang et al. reported that increased insular cortical thickness correlated with symptom severity in individuals with IGD. In another study, Lee et al. reported that subregions of the anterior cingulate cortex, another key node of the salience network, formed different functional connectivity patterns in subject with IGD with comorbid depression.

Problematic Internet game play is often accompanied by major depressive disorder (MDD). Depression seems to be closely related to altered functional connectivity (FC) within (and between) the default mode network (DMN) and salience network. In addition, serotonergic neurotransmission may regulate the symptoms of depression, including impulsivity, potentially by modulating the DMN. Hong et al. reported that the SS allele of 5HTTLPR genotype group showed greater FC within the DMN and salience network, and between these networks, compared to the SL + LL allele group. The results suggest that the short allele of 5HTTLPR may increase FC within the DMN and salience network, which may subsequently aggravate impulsive Internet game play in patients with MDD.

Kim and Kang investigated different reward systems implicated in IGD. For monetary reward, the IGD group exhibited stronger functional connectivity within the brain regions involved in motivational salience, whereas the group showed reduced functional connectivity the widely distributed brain areas involved in learning or attention. These differences in functional connectivity of reward networks, suggest that IGD is associated with an increased incentive salience or “wanting” process, which may serve as the neurobiological mechanisms underlying impaired goal-directed behavior.

Attentional bias toward addiction-related cues is also associated with incentive salience, but the pathophysiology of attentional bias in IGD is not well-understood, such as its relationship to compulsivity. Kim et al. used the electrophysiological marker of late positive potential (LPP) to compare attentional bias in IGD and obsessive compulsive disorder (OCD). Increased LPPs in response to disorder-specific cues (game-related and OCD-related) were found in both IGD and OCD groups, respectively. These results indicate that LPP is a candidate neurophysiological marker for cue-related craving in IGD and OCD.

Impairment of self-regulation is one of the major psychopathologies of addiction. Self-regulation ability is related to how well basic psychological needs are satisfied. These basic psychological needs, consisting of autonomy, competence, and relatedness, are important factors affecting individual growth and integration. Some individuals may rely on and overuse social media networks, as well as Internet games, in an attempt to meet basic psychological needs. Kim et al. investigated the neural correlates underlying the distorted self of individuals with IGD in relation to their satisfaction with basic psychological needs. Individuals with IGD had a negative ideal and actual self-image. Neurobiologically, dysfunction in the inferior parietal lobule associated with emotional regulation and negative self-evaluation was found in IGD. Recognizing that IGD often develops in adolescence, this self-concept problem should be noted and addressed with appropriate therapy approaches.

Neurobehavioral phenotypes are epigenetically controlled by non-coding RNAs including microRNAs (miRNAs). Since miRNAs can be detected in blood (plasma or serum), circulating miRNAs have a definite advantage as non-invasive biomarkers in neuropsychiatric disorders. Lee et al. identified IGD-associated miRNA markers by observing differentially expressed plasma miRNAs between the IGD and control groups. Through genome-wide screening of miRNA expression profiles and independent validation, three IGD-associated miRNAs (hsa-miR-200c-3p, hsa-miR- 26b-5p, and hsa-miR-652-3p) were discovered. Individuals with downregulation of all three miRNA are at high risk of IGD.

Autonomic nerve system (ANS) dysfunction has also been associated with substance abuse and behavioral addiction. As the ANS responds to internal and external stimuli to maintain homeostasis, its function is closely related to adaptive adjustments in behavior strategies. ANS dysfunction likely contributes to the development and maintenance of loss of control over gaming, as individuals with IGD are unable to adjust their behavior strategies despite negative outcomes. ANS function can be assessed non-invasively by measuring heart rate variability (HRV). Hong et al. demonstrated that individuals with IGD were characterized by reductions in high-frequency heart rate variability while the subjects were playing their favorite online game. Their results suggest that an altered HRV response to specific gaming situations is related to addictive patterns of gaming and may reflect the diminished executive control of individuals with IGD while playing Internet games.

As smartphone adoption and use has grown rapidly, there has been increased interest in the potential negative impact of excessive smartphone use. Chun et al. investigated altered brain connectivity associated with excessive smartphone use, and the relations between withdrawal symptoms, cortisol concentrations, and frontostriatal connectivity. They found that adolescents with excessive smartphone use had reduced functional connectivity in these regions related to cognitive control. Furthermore, Internet use withdrawal symptoms appear to elicit cortisol secretion, and this psychophysiological change may affect frontostriatal connectivity. These results provide important insights into the effects of excessive use of smartphones on brain functional connectivity in adolescence.

Gaming disorder and compulsive sexual behavior (CSB) disorder were recently included in the latest International Classification of Diseases (ICD-11). However, the WHO purposefully decided to classify compulsive sexual behavior disorder as an impulse control disorder, while gaming disorder was included to addictive disorders. Seok and Sohn found that individuals with problematic hypersexual behavior have diminished executive control and impaired functionality in the right dorsolateral prefrontal cortex, which is a core feature shared across both addictive disorders and impulsive control disorders. In addition, Gola and Draps reported that CSB is related to increased ventral striatal reactivity during the anticipation of erotic stimuli, in support of the theory of incentive salience. They suggested that further studies should be undertaken to examine neurobiological differences in these two disorders.

## Longitudinal changes of neurobiological correlates in behavioral addictions

This Research Topic also presents a series of novel studies that employ longitudinal designs, a design approach that historically has been quite limited in the IGD field. Lee et al.'s study aimed to identify the neuropsychological factors that promote improved recovery from IGD. They reported that individuals with IGD who had not improved at 6-month follow-up were more likely to have higher aggression and harm avoidance at baseline, indicating that gaming problems among these more complex cases appear less likely to resolve spontaneously. The assessment of aggression and harm avoidance levels may help predict the course of IGD.

Park et al. investigated neural connectivity associated with treatment responses in patients with IGD using resting-state electroencephalography (EEG) coherence analyses. Compared with healthy controls (HCs), patients with IGD showed increased beta and gamma intrahemispheric coherence and increased delta intrahemispheric coherence of the right hemisphere at baseline. After 6 months of outpatient management including selective serotonin reuptake inhibitors, patients with IGD exhibited improvements in IGD symptoms compared with baseline, but they continued to show increased beta and gamma intrahemispheric coherence compared with that of HCs. These findings suggest that significantly greater intrahemispheric fast-frequency coherence may be an important neurophysiological trait marker of IGD.

## Diagnostic and Treatment Approach

The final category of studies in this Research Topic involved neurobiological diagnostic and treatment approaches. Kim et al. investigated the relative value of behavioral, temperamental, and physical factors in predicting risk/problematic Internet use (ARPIU) in adolescents. They found that, among boys, severity of Internet addiction correlated inversely with the 2D:4D digit ratio and novelty-seeking, and positively with reward dependence scores when controlling for depression scores. These relationships were not found in girls, suggesting the need for gender-sensitive approaches to prevent ARPIU in youth.

The paper by Kim and Hodgins proposes a transdiagnostic treatment model of addictions that targets underlying similarities between behavioral and substance use addictions. Their model highlights various component vulnerabilities, each with intervention possibilities, including: lack of motivation, urgency, maladaptive expectancies, deficits in self-control, deficits in social support, and compulsivity. In another paper relevant to this topic, Blum et al. introduced the “Precision Addiction Management” (PAM)™, the customization of neuronutrient supplementation based on the Genetic Addiction Risk Score test result, along with a behavioral intervention. Finally, Bae et al. examined bupropion as a treatment modality for IGD and gambling disorder. Bupropion showed promise for improving problematic behaviors in both IGD and GD, however there were differing pharmacodynamics across the two groups.

In conclusion, the presented collection of original articles encompasses diverse research reports and review articles, with broad coverage of neurocognitive, neurophysiological, neurochemical, and neuroimaging research techniques. Together these articles demonstrate that the study of behavioral addictions from a neurobiological perspective is continuing to flourish and that there will be many exciting advances in this area that will improve our understanding, assessment, and treatment of individuals affected by these conditions.

## Author Contributions

All authors listed have made a substantial, direct and intellectual contribution to the work, and approved it for publication.

### Conflict of Interest Statement

The authors declare that the research was conducted in the absence of any commercial or financial relationships that could be construed as a potential conflict of interest.

